# Raman Study of the Diamond to Graphite Transition Induced by the Single Femtosecond Laser Pulse on the (111) Face

**DOI:** 10.3390/nano13010162

**Published:** 2022-12-29

**Authors:** Andrey A. Khomich, Vitali Kononenko, Oleg Kudryavtsev, Evgeny Zavedeev, Alexander V. Khomich

**Affiliations:** 1 Kotelnikov Radio-Engineering and Electronics Institute of the Russian Academy of Sciences, Vvedensky Sq. 1, 141190 Fryazino, Russia; 2 Prokhorov General Physics Institute of the Russian Academy of Sciences, Vavilov St. 38, 119991 Moscow, Russia

**Keywords:** diamond, femtosecond pumping, ablation, graphitization, Raman spectra

## Abstract

The use of the ultrafast pulse is the current trend in laser processing many materials, including diamonds. Recently, the orientation of the irradiated crystal face was shown to play a crucial role in the diamond to graphite transition process. Here, we develop this approach and explore the nanostructure of the sp^2^ phase, and the structural perfection of the graphite produced. The single pulse of the third harmonic of a Ti:sapphire laser (100 fs, 266 nm) was used to study the process of producing highly oriented graphite (HOG) layers on the (111) surface of a diamond monocrystal. The laser fluence dependence on ablated crater depth was analyzed, and three different regimes of laser-induced diamond graphitization are discussed, namely: nonablative graphitization, customary ablative graphitization, and bulk graphitization. The structure of the graphitized material was investigated by confocal Raman spectroscopy. A clear correlation was found between laser ablation regimes and sp^2^ phase structure. The main types of structural defects that disrupt the HOG formation both at low and high laser fluencies were determined by Raman spectroscopy. The patterns revealed give optimal laser fluence for the production of perfect graphite spots on the diamond surface.

## 1. Introduction

The unabated interest in the study of diamond is due to both the unique combination of its properties and the ability to reconfigure its atomic structure under external exposure, which makes it possible to design a material with desired properties at the nanoscale level if a suitable tool is available. Laser radiation is one of the most effective tools for locally transforming diamond into graphite.

The current trend in the laser processing of diamonds consists of the use of femtosecond laser pulses [1,2]. The mechanism of diamond graphitization significantly depends on the pulse duration. During a nanosecond-long laser pulse, the lattice can attain thermal equilibrium with the excited electrons, and the graphitization proceeds through a similar thermal process. However, when the duration of the pulse is reduced to the order of a femtosecond, there is no time for the electrons and lattice to come to an equilibrium, and the diamond–graphite phase transition is driven by the dense electron-hole plasma created by the laser [3]. Femtosecond radiation features an ultrahigh peak power, which provides strong nonlinear absorption and, accordingly, stable interaction with the structure of transparent targets. An ultrashort laser pulse enables the transmission of enormous power to the electronic subsystem of the crystal immediately on the optical table without using “megascience” installations, while tens of GW of optical power can be localized in a micron-size irradiated area. A strongly ionized and hot substance turns out to be completely dynamically amorphized, and its state is usually described as “warm dense matter” [4]. Since carbon has a large number of allotropes, relaxation from the “warm dense matter” state gives rise to a wide variety of carbon phases, from oriented crystalline graphite to exotic forms that cannot be obtained by other methods, for example, “Q-carbon” [5].

From a practical point of view, the ability to create graphitized conducting regions in a dielectric matrix is a unique feature of diamond that can be applied in a number of areas, including photonics, terahertz optics, electronics, etc. The development of this approach will lead to the implementation of the concept of a carbon composite matrix consisting of ordered conductive and dielectric components.

However, it should be noted that the degree of crystalline perfection of the laser-graphitized phase is rather low. The known studies are devoted to the irradiation of the (100) face of diamond. Raman microspectroscopy [6] and electron microscopy [7] identify the laser-modified layers on this face as a disordered form of carbon that is much closer to glassy carbon than to highly oriented pyrolytic graphite. Actually, laser-graphitized diamond is a mixture of amorphous carbon with predominant sp^2^ hybridization of bonds, which is mixed with nanocrystalline graphite with a grain size of ≈3 nm [8,9,10,11,12]. It is the high degree of amorphization that significantly deteriorates its conductivity (to 3–4 orders of magnitude below the conductivity of pyrolytic graphite) and, accordingly, narrows the range of possible applications.

Laser processing is considered the prime candidate for shallow junction and smaller device processing. The laser process formed irradiated areas with nearly identical shapes and sizes as the predesignated laser beam profile. The low structural quality of laser graphitized layers did not allow them to be widely used as electrodes and interconnects in diamond electronics. To form passive and active elements for diamond electronics (for example, those in which dopant incorporation and conductivity levels are controlled by manipulating laser irradiation [13,14]), layers with a highly ordered graphite-type structure are needed.

In a wide sense, the problem of controlling the electrical and optical properties of the laser-induced phase is solved by controlling the radiation parameters: the pulse energy, pulse duration, and wavelength. Here, we aim to achieve a particular and quite important task: an improvement in the perfection of the graphitic material produced with a laser. This possibility was predicted in [15,16] and was recently demonstrated experimentally [17]. It turned out that the orientation of the irradiated face of a diamond crystal has a decisive influence on the perfection of the resulting graphite. Since graphitization preferentially occurs through the transformation of three (111) planes of diamond into two (0001) planes of graphite, the stresses and strains in the graphite-like layer formed on the (111) face are minimal compared to those on the (100) and (110) faces [18].

The development of diamond-power electronic devices based on p–n junctions strongly relies on the ability to achieve efficient n- and p-type doping by phosphorus and boron, respectively. The (111) diamond plane has an eight times higher boron incorporation density [19] and at least two orders of magnitude higher phosphorus incorporation efficiency [20] than the (100) diamond lattice plane. The (111) diamond plane has also been found to be advantageous for the realization of superconductivity in diamond [21]. Due to the preferential orientation of the NV bond along the <111> direction [22], higher emission intensities and easier alignment of the magnetic field perpendicularly to the (111) diamond plane are observed [23].

Then, if the duration of an excitation pulse is significantly less than the transformation time of covalent bonds, the diamond lattice can be transformed into a graphite lattice with minimal dynamic and static distortions, which inevitably occur during the allotropic transformations and after them. In this work, we develop such an approach and apply Raman microspectroscopy to study the nanostructure and the structural perfection of the laser graphitized layer on the (111) diamond face in a wide range of laser fluencies. Besides searching for the optimal irradiation regime, the aim of the study was to establish the nature of structural defects associated with the degradation of the properties of laser-graphitized diamond.

## 2. Materials and Methods

The laser irradiation was carried out on the surface of a natural type IIa single crystal. The face (111) was obtained by mechanically splitting the original crystal, which resulted in numerous Walner lines on the cleaved surface. The width of large terraces in which the roughness reached the atomic level, and which were irradiated was ~100 μm. The nitrogen concentration in the sample did not exceed 10^17^ cm^−3^ and measured photoluminescence spectra did not contain any impurity–defect band. The only noticeable line with a maximum at 492 nm [24] and FWHM of ~3.5 nm had an intensity 5000 times lower than the Raman diamond peak (see Supplementary Materials).

To initiate graphitization, we used the 3rd harmonic of a Ti:Al_2_O_3_ laser with a Spectra Physics regenerative amplifier. The pulse duration was 120 fs at half maximum. It is known that the duration of femtosecond pulses is comparable to the period of lattice oscillations, and therefore the pulse energy is transmitted faster than the possible lattice rearrangement. Under these conditions, the trailing edge of the pulse does not interact with the material modified by the leading edge of the pulse, i.e., the effect of laser radiation on the graphitized phase is excluded. The radiation wavelength was 266 nm; the use of UV radiation provided strong absorption near the diamond surface (direct two-photon band-to-band transition). In addition to effective excitation of the electronic subsystem of diamond, UV pulses minimize the heating of plasma due to the relatively weak inverse Bremsstrahlung absorption.

The irradiation pulse energy ranged from the graphitization threshold to a maximum value of about 10 μJ. So, the dynamic range exceeded 20. The laser beam was focused on the surface of the sample by an aspherical lens with a focal length of 35 mm. The aperture of the focused beam was about NA = 0.1. The intensity distribution on the surface was close to a Gaussian distribution with a beam radius of ≈2.21 μm at the 1/e level. The resulting laser fluence in the center of laser spot varied within F ≈ 1–45 J/cm^2^. The size of graphitized microcraters on the surface of the laser-modified diamond ranged from 0.5 to 8 µm.

The diamond surface profile in the irradiated region was monitored by white light interferometry and atomic force microscopy. The measurements were carried out both after laser irradiation and after annealing in air at 600 °C, thus completely removing the sp^2^ phase from the surface.

To identify carbon phases that arise on the diamond at moderate and high levels of laser damage, the confocal Raman spectroscopy technique was used. The Raman spectra were measured on a Horiba Jobin Yvon LabRAM HR800 spectrometer (Lille, France) with excitation at 473 nm. It is noteworthy that to characterize laser-graphitized diamond, the Raman spectra are usually limited to the range of 1000–1800 cm^−1^, showing the D and G bands of the graphitic phase. Here we expanded the spectral range up to 200–2300 cm^−1^. This significantly enhances the informativeness of the Raman spectra, as we demonstrated recently in the study of structural defects and the features of the diamond–graphite transition in radiation-damaged diamonds [25]. An Olympus objective (magnification ×100, NA = 0.95) was used for focusing on the spectrometer, and the diameter of the laser spot was approximately 1 μm. Translation over the surface inside the graphitized spots with a step of 0.5 μm was performed using a motorized stage (Märzhäuser Wetzlar GmbH, Wetzlar, Germany) with the measurement of the complete Raman spectrum at each point of the scan. Analysis of these spectra made it possible to trace in detail the changes in the structure of laser-modified diamond in a wide range of laser fluencies.

## 3. Results and Discussion

### 3.1. The Main Principles of Laser Graphitization and Ablation under Femtosecond Irradiation

**Figure 1:**
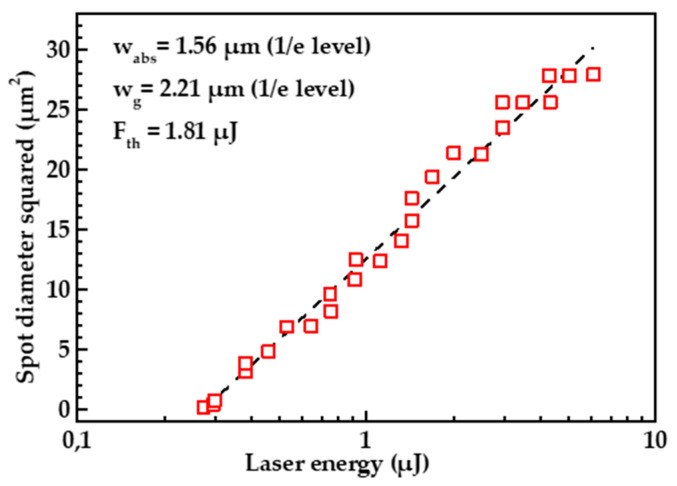
Dependence of laser spot diameter squared on the pulse energy. Gaussian beam radius taken at 1/e level and laser graphitization threshold is denoted on the plot.

Figure 1 demonstrates the diameter of a crater formed on the diamond surface due to the femtosecond pulse irradiation as a function of the pulse energy. Since in the given coordinates (the square of the laser crater size–the logarithm of energy), this dependence is very close to a linear function, the absorbed energy profile is close to a Gaussian one: $F\left(r\right)=F_{0}\times exp\left(-r^{2}/w_{abs}^{2}\right)$, where F is the local fluence of absorbed energy; r is the distance from the center; $F_{0}=F\left(0\right)$ and $w_{abs}$ is the Gaussian radius of the absorbed energy profile, which shows at what distance from the center the amount of absorbed energy drops e times. The parameter $w_{abs}$ calculated from the above dependence was $w_{abs}=1.56$ μm.

Taking into account the two-photon nature of absorption (the photon energy is 4.65 eV, and the diamond band gap is 5.4 eV), we can calculate the Gaussian radius of the laser beam, $w_{g}=\sqrt{2}w_{abs}=2.21$ μm, and the threshold laser energy density $F_{0}^{th}=1.81\;J/{\mathrm{cm}}^{2}$ at which graphitization develops on the surface.

**Figure 2:**
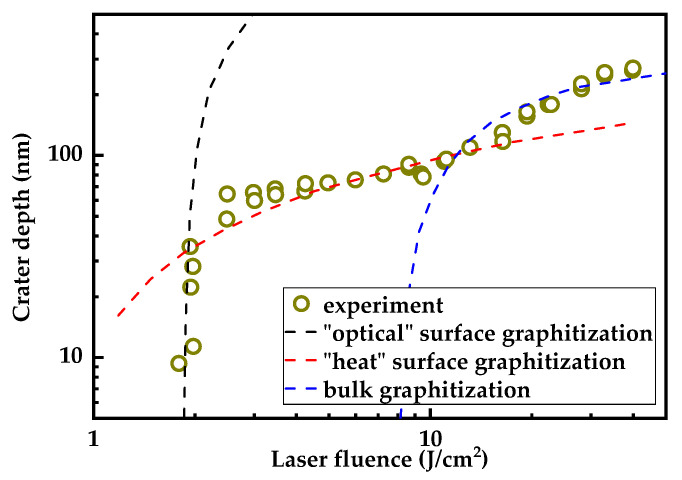
Dependence of ablated crater depth measured after oxidation of graphitized layer on the laser fluence. Curves exemplify different regimes of surface and bulk graphitization induced by femtosecond radiation.

Figure 2 shows the experimental depths of the ablation craters as a function of the laser energy density. The measurements were carried out after oxidizing the sample in an oven (600 °C) and removing the sp^2^ hybridized phase from the bottom of the craters (see Supplementary Materials).

In fact, the measured crater depth (Figure 2) determines the distance that the graphite phase moves into the bulk of the crystal. It is clearly seen that the dependence is complex, which, in our opinion, can be explained by considering three different regimes of the graphitization of the diamond surface under single-pulse laser irradiation.

First of all, it should be noted that at present it is not quite clear how much the equilibrium and nonequilibrium mechanisms contribute toward laser graphitization. There are two different possibilities. The first is nonequilibrium graphitization, which occurs during a very short period while the recombination of electron–hole pairs excited by radiation takes place (~30–100 ps [26]). In this case, the lattice transformation is attributed to numerous (~10^20^ cm^−3^ and more) breaks of covalent bonds caused by the transitions of carriers from the states localized in bounds to the quasi-free states. With an increase in the pumping energy, this process leads to the so-called “Coulomb explosion” [27]. On the other hand, the recombination of excited carriers releases energy that is ultimately transmitted to the phonon subsystem of the crystal. As is well known, the same process—the rearrangement of the lattice—results from the significant heating of the lattice. Fortunately, both these mechanisms have similar properties in terms of the development of graphitization: (i) they have a quasi-threshold character, (ii) the degree of heating and the degree of ionization equally depend on the irradiation parameters, and (iii) they are subject to diffusion spreading. Due to this similarity, the ablation dependence (Figure 2) can be analyzed from general considerations with regard to the initial distribution of the absorbed optical energy, its diffusion, and the dynamics of the graphitization front.

At energies close to the threshold, the first regime is realized, in which the depth of graphitization is determined by the penetration depth of the optical field. For two-photon absorption, the depth of graphitization can be described by a simple relationship:

(1) $$D\left(F\right)=\frac{\tau }{\beta }\left(\frac{1}{F^{th}}-\frac{1}{F}\right),$$

where τ is the pulse duration and $\beta \approx 10^{-9}\;\mathrm{cm}/W$ is the two-photon absorption coefficient of diamond (see Supplementary Materials). Under our conditions, the field penetrated the crystal by ~1 μm, and Equation (1) describes well the initial sharp increase in the thickness of the graphitized layer from ~10 nm to ~70 nm (black curve, Figure 2). Note, that this regime can be considered as nonablative graphitization [17,28], i.e., the regime at which the graphitization takes place while the ablation does not. The profiles of the laser spots support this view, they looked swollen and did not contain any specific vaporization trace on the surface.

However, at energies just above the threshold, a transition to the second graphitization regime, which is characterized by strong saturation, is observed. Starting from ~70 nm and up to ~100 nm, the depth of graphitization grows slowly, according to a law close to logarithmic (red curve, Figure 2). In our opinion, the key role in this regime is played by the surface from which the transformation of sp^3^-hybridized bonds into sp^2^-hybridized bonds starts. This is true for both the regimes involved, just as for the thermal graphitization [15,16]. In fact, in these regimes, one should think of a graphitization wave. The surface is the starting point for this wave. Calculations confirm this scenario and show that the lattice rearrangement occurs layer by layer at a typical rate of 1 atomic layer (~0.2 nm) per 0.1 ps [15]. The continuous nature of graphitization in these regimes is apparently the reason for the saturation of its rate at moderate fluencies. Diamond has a record thermal diffusivity ($\chi_{g}∼10\;{\mathrm{cm}}^{2}/s)$, and the subsurface layers have time to cool down before the graphitization wave reaches them. In this case, the heat released in the region of already graphitized material (closer to the surface) turns out to be “locked,” since the thermal diffusivity of graphite is much lower than that of diamond ($\chi_{g}∼0.1\;{\mathrm{cm}}^{2}/s)$. As a result, the increase in the laser energy causes the steadily incremental superheating of ablated surface carbon.

Finally, when the laser fluence exceeded a certain threshold $\left(10\;J/{\mathrm{cm}}^{2}\right)$, bulk graphitization started in the crystal, which did not initiate at the surface but occurred simultaneously in the entire excited volume (~1 μm). This process was accompanied by a strong expansion of the graphitized phase due to the difference in its specific mass density to that of a diamond. The surface swelling in the laser spot reached ~0.5 µm, indicating high dynamic stresses in the bulk graphitization region. In this regime, the dependence of the graphitization depth on the laser fluence is very well described within the model of optical field penetration into diamond and two-photon absorption (blue curve, Figure 2). The threshold of bulk laser graphitization, which was an adjustable parameter in Equation (1), was $8\;J/{\mathrm{cm}}^{2}$.

The thickness of the layer graphitized by laser irradiation was estimated by the well-known technique based on the attenuation of the intensity of the diamond line in the Raman spectra due to the linear absorption of exciting and scattered radiation in the graphitized layer [29]. This method proved effective for nondestructive testing of the thickness of graphitized layers up to 300 nm [1,29,30]. Its main drawback (just as the drawback of other nondestructive optical methods) is associated with the possibility of interference beats of the exciting beam intensity. The value of the absorption coefficient at a wavelength of 503 nm (the wavelength of the diamond peak) was taken equal to 1.16 × 10^5^ cm^−1^ [31]. This value is typical for nanocrystalline graphite.

**Figure 3:**
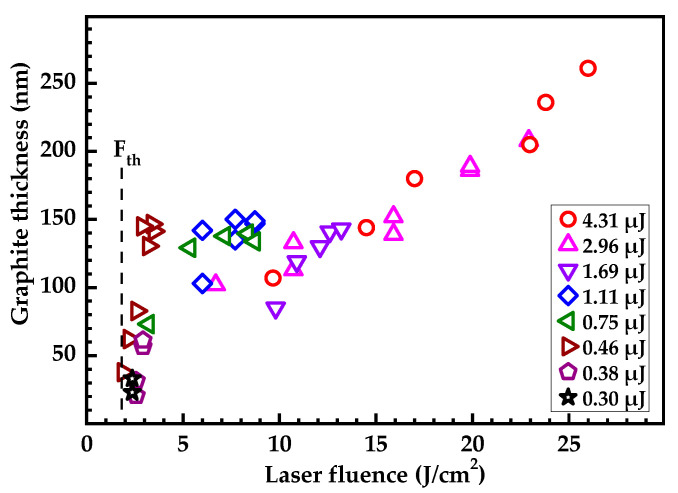
Thickness of graphitized layer deduced from intensity of Raman diamond line versus the laser fluence. F_th_ denotes graphitization threshold. Legend denotes the energies at which the spots explored were produced.

The thickness of the graphitized layer as a function of the laser fluence is shown in Figure 3. The data presented combines the results of measurements performed at different points of laser spots produced at different energies. The absolute values of the thickness of the graphitized layer are in reasonable agreement with the depth of propagation of the graphitic front (Figure 2), taking into account the change in the specific density of the diamond during graphitization (see Supplementary Materials). Moreover, there is a clear correlation between these values, despite the significant scatter of thickness data due to both the above interference instability and the inhomogeneity of the sp^2^ phase structure over the layer [17]. This correlation is expected since the given data describe the same process of the penetration of graphite into diamond under single-pulse irradiation.

### 3.2. Correlation between Graphitization Regimes and the Structure of Raman Spectra in the Region of D and G Lines

All carbon substances have common features in their Raman spectra. These are the so-called G and D peaks with maxima at 1580 and 1350 cm^−1^, respectively [32]. The spectral position, intensity, and FWHM of these peaks are used to describe the structure of sp^2^ carbon materials. These bands have different natures. The G-band is a doubly degenerate (TO and LO) phonon mode (E2g symmetry) at the center of the Brillouin zone. In the presence of defects in sp^2^ carbon materials, the Raman spectrum always contains the D band. The origin of the D peak in carbon materials is well-established to be related to defect-induced double-resonant scattering [33,34], so its spectral position and FWHM depend not only on the concentration and types of defects but also on the laser wavelength in Raman measurements. The double-resonance model provides a deep understanding of the most experimental observations of the D peak. Within this model, the defect-induced Raman process consists of four virtual transitions: (i) a laser-induced excitation of an electron/hole pair through the energy gap between the conduction and valence bands at the k point; (ii) inelastic scattering of the excited electron by a phonon with an exchanged momentum q that is close to the k point of the graphite Brillouin zone; (iii) elastic scattering of an electron by a lattice defect; and finally, (iv) electron/hole recombination with the conservation of k [35]. Obviously, the second and third steps in these transitions can be interchanged. The D peak is activated during an inter-valley process, whereas another disorder-induced peak, the D′ band at ~1620 cm^−1^, originates from a double-resonance intra-valley process connecting two points in the same Dirac cone around the k (K′) point [36].

**Figure 4:**
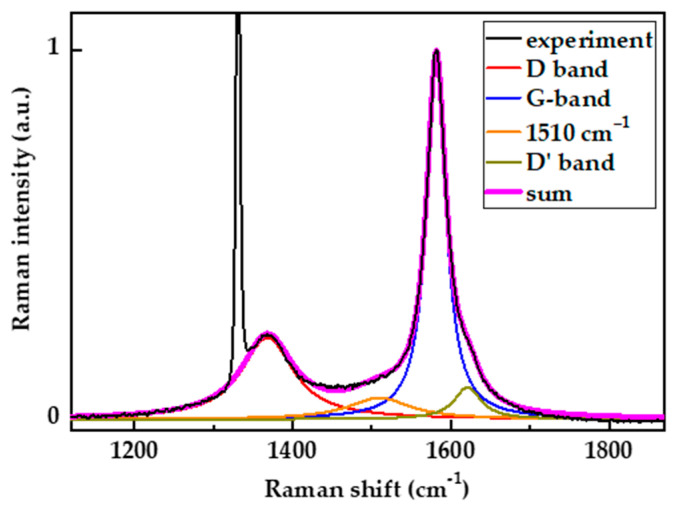
An example of the decomposition of the Raman spectrum into the Lorentz and Gaussian contours.

Figure 4 shows the decomposition of the typical Raman spectrum measured from the diamond surface irradiated by a single pulse of the femtosecond UV laser. The use of the Breit–Wigner–Fano (or pseudoVoigt) profile turned out to be suitable for subtracting the diamond peak from the Raman signal. The Breit–Wigner–Fano profile most correctly describes the slight asymmetry of the diamond band resulting in mechanical stresses arising at the diamond–graphite interface in the laser crater [37,38]. To fit the rest of the Raman spectrum, the four-band decomposition procedure [39] was used, namely G, D, and D′ bands and the band between G and D bands at ≈1500 cm^−1^. The need for such a band for the fitting of Raman spectra of nanocrystalline graphite was first proposed by Rouzaud et al. [40]. The band at ≈1500 cm^−1^ is sometimes called the “A band” [39]. This band was interpreted as “out-of-plane defects”, which are of sp3 hybridization [41,42]. The A-band also can be ascribed to a double resonance with the iTO phonon branch [43]. The G, D, and D′ bands were fitted by Lorentzian and the A band by Gaussian. As is evident in the data in Figure 4, the agreement between the results of decomposition and measurement is quite close over the entire spectral range.

Figure 4 exemplifies the Raman spectrum of the laser spot produced at a low laser fluence. The spectrum given is typical for ordered graphite and contains two distinct peaks near 1365 cm^−1^ and 1585 cm^−1^, suggesting that the graphitic phase is composed of relatively large clusters with only limited damage to the crystalline structure. The full width at half maximum (FWHM) of the G line was equal to 30 cm^−1^. The intensity of the D line was noticeably lower than the intensity of the G line. The ratio I_D_/I_G_, which is used to determine the size L_a_ of a carbon cluster in the spectra of carbon materials in the low laser fluence graphitization region achieved 0.2–0.3.

**Figure 5:**
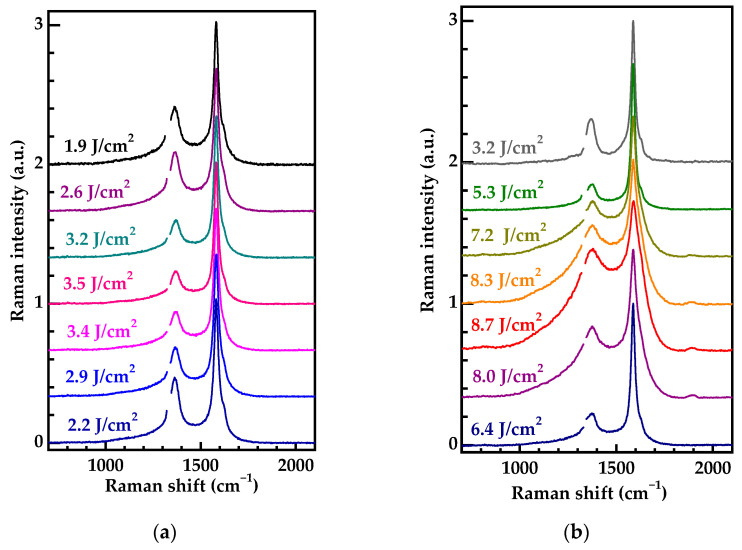
Raman spectra of graphitized structures: obtained at a laser pulse energy of 0.46 μJ (**a**) and 1.11 μJ (**b**). The spectra are normalized to the amplitude of G-band and shifted for clarity.

Figure 5 shows a set of spectra that were obtained by scanning two craters and normalized to the amplitude of the G-band. The first crater was produced with 0.46 μJ pulse energy (Figure 5a). As is evident in the data, the D-band exhibits a clearly defined behavior. When moving from the edge to the center of the crater, the D-band intensity gradually falls and reverses to the almost same value when moving to another edge. In other words, the higher the laser fluence is, the better diamond is produced. It has to be emphasized that such a dependence was rather unexpected. It contradicts the pattern which we observed previously [17] and, as we will see later, results from the low-fluence regime of the graphitization.

The corresponding change in the ratio of the intensities of the D and G-bands (I_D_/I_G_) varied from 0.40 to 0.23 within 0.46 μJ laser spot. The size L_a_ of a carbon cluster can be written as [44]:

(2) $$L_{a}\left(nm\right)=\frac{560}{E_{laser}^{4}}{\left(\frac{I_{D}}{I_{G}}\right)}^{–1},$$

where E_laser_ is the photon energy for the pumping laser (473 nm) expressed in electronvolts. Thus, within the crater, the given L_a_ increases from ~30 nm at the periphery to ~52 nm at the center. Note, that the FWHM of the G-band changes slightly, decreasing from 30 cm^−1^ at the periphery to 27 cm^−1^ in the center of the crater. Such FWHM values of the G-band are typical for HOG.

The trend of change in the structural perfection of the graphitized phase in the 1.11 μJ spot was reversed (Figure 5b) and coincided with that what was observed earlier [17]. Moreover, the increase in the local laser fluence resulted in the significant broadening of the D- and G-bands, thus indicating the gradual disorder of the HOG phase. The closer one got to the spot center, the higher the disorder degree was. It is noteworthy, that at the spot edge where the laser fluence was low (from 3.2 to 5.3 J/cm^2^), the structural perfection returned to the trend found for the nonablative graphitization.

This suggests that, first, the structural perfection depends explicitly on the local laser fluence, and second, that there is a certain laser fluence where the structural perfection achieves a maximum. It is of interest that this maximum can be located inside a single laser spot like it was found for the 0.46 μJ and 1.11 μJ spots (Figure 5). A similar improvement in the structural perfection of the graphitized layer with an increasing laser fluence above the graphitization threshold was observed when single femtosecond laser pulses were applied to the (111) surface of diamond irradiated with fast neutrons [45]. Such an effect in fast neutron irradiated diamonds could be partly explained by the competition between laser annealing of radiation-damaged diamond and its surface graphitization.

**Figure 6:**
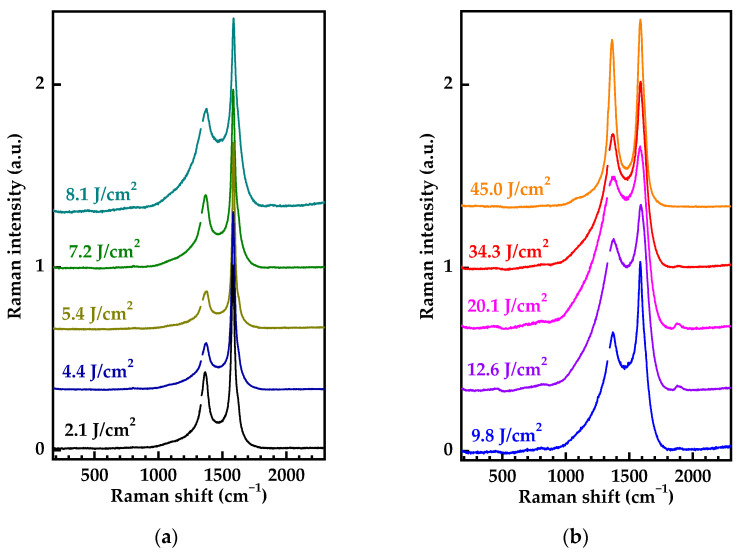
Raman spectra of graphitized structures obtained at lower (**a**) and higher (**b**) laser fluences. All the spectra are normalized to the amplitude of G-band and shifted for clarity.

Figure 6 displays the Raman spectra of the same samples in the 200–2300 cm^−1^ spectral range over a wider range of laser fluences. The shape and parameters of the spectra measured on different spots at close values of the laser fluence differed insignificantly. All the trends that were observed when scanning across graphitized spots (Figure 5) are reproduced in Figure 6, including the structural perfection at a laser fluence above the graphitization threshold and broadening of the D- and G-band in the laser fluence range from 7 to 20 J/cm^2^. Synchronously with the broadening of D- and G-bands, additional low-intensity bands with maxima at 455, 810, and 1890 cm^−1^ appeared in the Raman spectra. These bands are discussed in more detail in the third section of the article. The narrowing of D- and G-bands at laser fluences above 20 J/cm^2^ (Figure 6) is due to the processes of bulk diamond graphitization.

**Figure 7:**
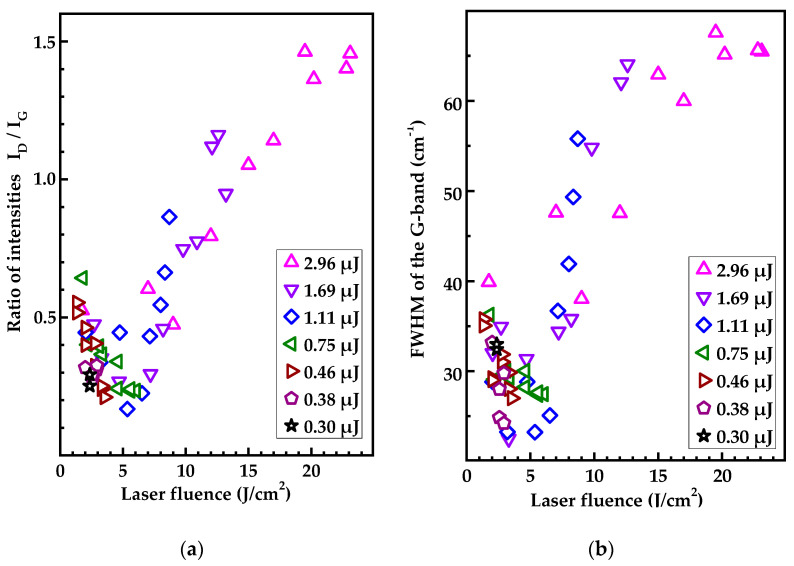
(**a**) The ratio between intensities of the D- and G- bands and (**b**) FWHM of the G band in the Raman spectra versus the laser fluence. Legends denote the pulse energies at which the spots explored were produced.

With increasing laser fluence starting from 6 ÷ 7 J/cm^2^, the FWHM of the G band increases (Figure 6a and Figure 7), arising from the disordering of the graphitized layer, which should result in deterioration of its electrical properties. Since overheating and ablation of the material during the development of surface graphitization completely destroys the ordered structure, all the potential advantages of laser irradiation of diamond (UV radiation, a single femtosecond pulse, and an (111) oriented diamond face) cease to be significant, and the structure of the graphitized layer differs insignificantly from the results obtained in other studies.

The main parameters of the Raman spectrum, which characterize the ordering degree of graphite, are the width of the G band and the ratio I_D_/I_G_ of the amplitudes of the D and G bands. These parameters are shown as a function of the local laser fluence in Figure 7. We can see that the minimum FWHM of the G band ranges from 20 to 30 nm, which is comparable with the same value for pyrolytic graphite [46]. At the same time, the ratio I_D_/I_G_ does not exceed 0.20 even at extreme points, and its average value in the minimum region (4–6 J/cm^2^) is about 0.25, which corresponds to a graphite nanocrystallite size of L_a_ ≈ 60 and 50 nm, respectively. Thus, even under optimal irradiation conditions, the spectra contain a pronounced D band, which, as is commonly believed, reflects the degree of defectiveness of the graphite.

The data in Figure 7 point to a number of significant trends. First, the most perfect graphite is formed at the laser fluence of 4–6 J/cm^2^, which is noticeably higher than the graphitization threshold. Second, at a higher laser fluence the degree of disorder in the graphitized material rapidly increases. Moreover, at a laser fluence above 6 J/cm^2^, this disordering has an abrupt character. During bulk graphitization, the FWHM of the G band shows a significant decrease from 100 cm^−1^ in maxima down to 60 cm^−1^ (not shown here). More detailed data on the structure of the graphitized material in the bulk graphitization regime will be given in a separate publication.

We emphasize that since the thickness of the graphitized layer sharply increases with increasing the value of the laser fluence (to 100 nm and higher), this may somewhat distort the real picture. Actually, Raman spectroscopy characterizes mainly the upper layer (up to 200 nm), which is easily penetrated by the exciting radiation and is responsible for the main Raman signal. Under these conditions, the structure of the layers located in the bulk near the graphite–diamond interface is less pronounced in the spectra (since the diamond peak is visible in all spectra, this means that the Raman signal is formed over the entire depth of the graphitized layer).

As for the optimal structure of the graphitized layer, under the experimental conditions it is achieved at a laser pulse energy of 0.75 μJ (Figure 7); the laser fluence at the center of the spot was 6 J/cm^2^. This laser graphitization regime leads to the formation of a material close to HOG with almost uniform thickness over the entire area of the spot (see Supplementary Materials).

The detailed studies carried out make it possible to pose for the first time the problem of finding a correlation between the degree of ordering of the obtained carbon phase and the above-described regimes of laser graphitization. It is important to emphasize that, according to the calculations, the diamond–graphite transition proceeds continuously, passing successively through various stages of disordering of the diamond lattice and alignment of the graphite lattice [15,16]. The pulsed excitation of the electron and phonon subsystems followed by their rapid relaxation leads to the “freezing” of the rearranged structure, which makes it possible to trace the various stages of the transition. These data clearly show that the structure of the graphitized layer is quite clearly determined by the physical features of the dissipation of optical energy in diamond.

First, the maximum perfection of a graphite-like (“graphitic”) lattice found in this work at energy densities above the threshold not only allows one to choose the optimal regime of laser irradiation, but also points out the most important aspect of the laser-induced diamond–graphite transformation, namely that laser graphitization is only partly a threshold process. In fact, the perturbed diamond lattice, having passed a certain stability limit, evolves smoothly to a certain extent. By changing the laser irradiation level, one can control the state of the structure, obtaining “under-graphitized” diamond, “perfect” graphite, and “damaged” graphite.

Second, the damage mode, in which the structure of the graphite phase becomes a nanocrystalline, is obviously associated with surface overheating, which develops in the regime of surface ablation. As discussed further, even if the resulting layer can be considered as a crumpled HOG under near-threshold irradiation, its structure will inevitably collapse with an increasing temperature, forming partially mutually oriented nanocrystals.

### 3.3. Specific Features of the Raman Spectra of the Phase Graphitized on the (111) Surface in the Low- and High-Frequency Ranges

**Figure 8:**
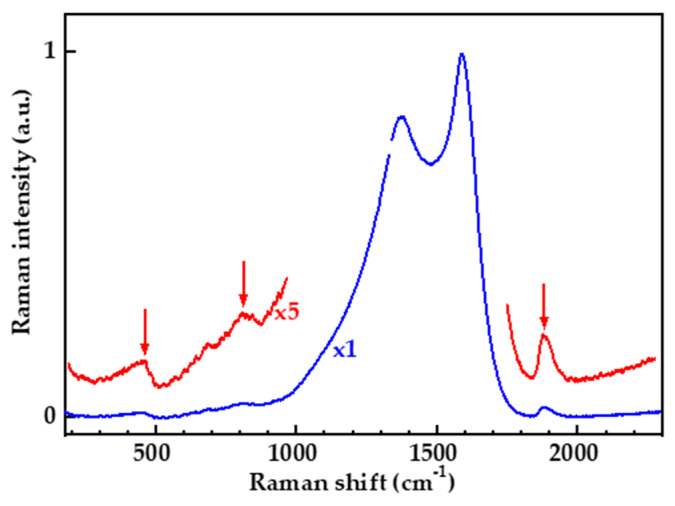
Raman spectrum of graphitized structure obtained at a laser fluence 20 J/cm^2^ (blue line), and the fragments of the same spectrum, multiplied five times (red line). The arrows indicate the bands at 455, 810, and 1890 cm^−1^.

In the Raman spectra of laser-graphitized (111) diamond in addition to D, A, G, and D′ bands, we revealed bands with maxima near 455, 810, and 1890 cm^−1^ (Figure 8). The maximum intensity of these bands did not exceed 1–2% of the intensity of the G-band.

Traditionally, the main effort in interpreting the Raman spectra of the laser-graphitized diamond concentrated on the range above 1000 cm^−1^ and we could not find any data outside this range. The bands given were not discussed earlier, most likely because of the quite low scattering intensity. The reason for why the numerous studies of the laser graphitization did not detect the low- and high-frequency bands is unclear. We cannot rule out the significance of the single ultrashort pulse irradiation applied here. The complete absence of background photoluminescence in the spectra of the crystal may also play a role. We cannot unambiguously state that the bands with maxima around 455 cm^−1^, 810 cm^−1^, and 1890 cm^−1^ are specific for graphitization of the (111) diamond face. However, it should be noted that, as far as we know, the 810 cm^−1^ band was only mentioned in the study of pyrolytic graphite together with some other quite weak Raman lines [47].

The nature of low-frequency bands in carbon materials remains controversial [48]. The bands at 455 and 810 cm^−1^ may be due to double-resonance processes, since the double-resonance model was also applied to phonons belonging to all six branches of the graphite Brillouin zone. According to theoretical calculations, within the framework of the double-resonance model, one can expect the manifestation of low-intensity bands in the low-frequency region of the Raman spectra of defective graphene, which, according to the nomenclature proposed in [34], are called D3, D4, D5, and D” bands. In this case, the D3 and D4 bands associated with phonons near the Г point of the Brillouin zone have a momentum very close to the momentum of the phonons associated with the D′ line, and the bands associated with phonons near the k point of the D5 and D” bands have a momentum very close to the momentum of the D phonons. According to [34], the D3, D4, D5 and D” bands are much weaker than D and D′, since the electron–phonon coupling (between π-electron bands) for these branches is much weaker than that for the D and D′ bands [49]. The position of the D3, D4, D5 and D” bands depends on the wavelength of the laser used in the measurements of the Raman spectra. In the case of a laser with a wavelength of 473 nm, these bands would have been observed at 310, 505, 840 and 1210 cm^−1^ [34], which does not coincide with the spectra shown in Figure 8.

The spectral position of the bands at 455 and 810 cm^−1^ also does not coincide with the features in the spectra of the phonon density of the states of diamond [50] and graphite [51] but is in good agreement with the vibrational density of state calculations performed using the “melt and quench” scheme via molecular dynamics simulation in [52]. The 2-D amorphous graphene (a-G) is an idealized structure consisting of pentagons and heptagons in addition to hexagons with no coordination defects and a predominant sp^2^ bonding [53]. Amorphous graphene, for example, can be obtained by the exposure of a crystalline/pristine graphene sheet to an electron beam to produce an amorphous monolayer [54]. Similarly, 3D a-G has been modeled in [52], by introducing 5- and 7-membered rings with a Wooten–Weaire–Winer scheme [55]. The 3-D amorphous graphene consists of a three-dimensional network of curved graphene sheets with a small percentage (11%) of sp3 hybridized carbons connecting the sheets and a few sp^1^ hybridized carbons [52]. Evidence in support of the curved graphene models has been obtained from TEM images of very thin structures [56]. In [52], using ab initio methods, the authors calculated the vibrational density of states for 3-D amorphous graphene. It shows two distinctive components: a sharp peak near 814 cm^−1^, a broader peak at 1300 cm^−1^, and a narrow neck at 470 cm^−1^. The spectral dependence of the vibrational density of states for 3-D amorphous graphene agrees well with our measurements of the Raman spectra (Figure 8).

The observation of band characteristics of the vibrations of curved sheets in 3-D amorphous graphene in the Raman spectra of laser-graphitized diamond is in agreement with the ideas about the transformation of diamond under intense laser irradiation. Based on the data demonstrated, we believe that graphitized layers obtained at the laser fluences of 4–6 J/cm^2^, which are optimal from the viewpoint of structural perfection, can be considered as folded multilayer graphene rather than as nanocrystalline graphite. Folding is a fundamental and apparently unavoidable aspect of the transformation of the (111) layers of the diamond lattice into the (0001) layer of the graphite lattice. The reason for folding is that the area density of the atoms in the (111) plane of the diamond lattice is approximately 1.08 times greater than that of the graphene sheet. Therefore, the graphene layers on the diamond plane should be compressed, and since they cannot, they are crumpled into folds. First, such folds and the associated tensile stresses on the bends explain the significant broadening of the G band compared to the HOG, the strong unpredictable jumps in its position [57], and the appearance of the D band even on the thinnest of the laser-graphitized layers [58]. Second, the strength of the crumpled sheet is significantly less than that of the flat one, and the formation of thermally stimulated defects and ruptures in it is much more probable. The question of minimizing the residual heating and avoiding damage to the crumpled graphene remains open. The question of the influence of folds on the transport and thermophysical properties of layers formed by radiation also remains open.

**Figure 9:**
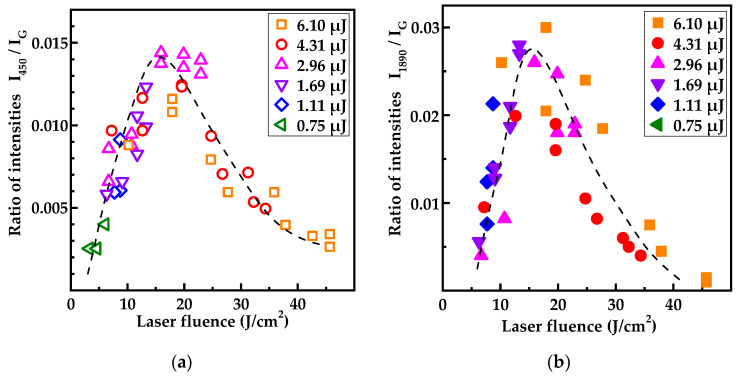
The ratio between intensities of the various bands to the G peak in the Raman spectra: (**a**) 455 cm^−1^ band; (**b**) 1890 cm^−1^ band. Legend denotes the energies at which the spots explored were produced. Dotted curves shown as eye guides only.

The fluence dependence (Figure 9b) of the band with a maximum at 1890 cm^−1^ and a half-width of ~45 cm^−1^ (Figure 8) is at first glance similar, but in reality, differs from the dependence of the bands at 455 and 810 cm^−1^ (Figure 9a). In terms of frequency, the 1890 cm^−1^ band is outside the spectral range where the first-order vibration frequencies are found in diamond [50] or graphite [51]. The several defect-induced and second-order double-resonant modes can be observed in graphite and other forms of sp^2^-bonded carbon. The 1890 cm^−1^ band (Figure 8) coincides in spectral position with the second-order double-resonant mode for the combination of the in-plane transverse optic (iTO) and longitudinal acoustic (LA) modes (iTO-LA [59]) if a 473 nm laser is used for Raman spectra measurements. In this case, an asymmetric peak consisting of two modes, named iTA-LO [60] and LO-LA [61] with a maximum near 2075 cm^−1^, should also be observed in the Raman spectrum. However, a peak near 2075 cm^−1^, as well as a peak at 310 cm^−1^ corresponding to the first-order double-resonant mode of the LA phonon, are absent in the Raman spectra (Figure 9). This allows us to state that the band at 1890 cm^−1^ (Figure 8) is not due to the second-order double-resonant mode.

In the Surface Enhanced Raman Scattering (SERS) spectra of carbon films produced by conventional ns pulsed laser deposition [62] was observed intense band with maxima at about 1900 cm^−1^ and the same spectral shape as the 1890 cm^−1^ band in Figure 8. The authors of [62] hypothesized that the band observed in the SERS spectra is due to vibrations of sp-carbon-atom wires embedded in or mixed in mainly sp^2^ amorphous carbon films. Linear carbon chains can form as an intermediate [63] phase during the transformation of diamond into graphite, but under normal conditions they show low stability. Additional studies are needed to clarify the nature of the 1890 cm^−1^ band.

## 4. Conclusions

We have studied the process of graphitization of the (111) face of a diamond by a single femtosecond pulse with energy ranging from the diamond graphitization threshold of ~2 J/cm^2^ to the air breakdown threshold of ~20 J/cm^2^ and higher. Using the Raman spectroscopy data, we traced the evolution of the efficiency of laser graphitization and the evolution of the crystal structure of the graphitized layer with increasing laser energy. We have found that, depending on the irradiation energy, three regimes of femtosecond graphitization of diamond are realized: (1) for energies close to the threshold, the graphitization rate is determined by the penetration of the optical field into the crystal; (2) with an increase in the irradiation energy, the depth of penetration of the graphitization front abruptly saturates, which is attributed to the limitation of the graphitization wave velocity; (3) and when the laser energy density exceeds a certain threshold, bulk graphitization is initiated in the crystal volume, which is not associated with the surface but occurs simultaneously in the entire excited volume (~1 μm).

We have established a clear correlation between the graphitization regimes and the structure of the graphitized phase. We have found that the laser-induced layer obtained near the graphitization threshold is sufficiently ordered and have proposed a model that describes this layer as a highly oriented graphite consisting of strongly crumpled graphenic sheets. We consider the detected 455 and 810 cm^−1^ Raman bands as a manifestation of the amorphized structure of graphenic sheets produced.

The degree of HOG ordering was found to have a distinct maximum at the laser fluences of ~4 ÷ 6 J/cm^2^. With a further increase in the laser’s fluence, overheating and ablation of the material completely destroyed the ordered structure. The regularities revealed new possibilities for controlling the properties of a graphite-like layer on diamond and make it possible to optimize laser irradiation in terms of increasing the crystalline perfection of graphite and the amount of the synthesized conducting phase.

## Data Availability

Additional data are available upon request to the corresponding authors.

## Supplementary Materials

The following supporting information can be downloaded at: https://www.mdpi.com/article/10.3390/nano13010162/s1, Figure S1: rocking curve of the diamond crystal used for TRS-K double-crystal monochromator; Figure S2: photoluminescence spectrum of the natural diamond (111) plate under study; Figure S3: (a) cross profile of an ablation crater with a graphitized structure obtained at a laser pulse energy of 0.75 μJ after removal of the sp^2^ phase. The dots indicate the projections of the spots where the Raman spectra were recorded. (b) Raman spectra at various points of graphitized layer obtained at a laser pulse energy of 0.75 μJ. The colors of the spectra correspond to the color of the dots in figure (b); Figure S4: An example of the decomposition of the Raman spectrum with Voigt profile of diamond peak [24,29,31].
