# Supplementary material for: Raman Study of the Diamond to Graphite Transition Induced by the Single Femtosecond Laser Pulse on the (111) Face
Source: Nanomaterials (Basel). 2022 Dec 29;13(1):162. doi: 10.3390/nano13010162 (PMC9824279; doi:10.3390/nano13010162)
Supplement: Supplementary file 1 [file nanomaterials-13-00162-s001.zip › nanomaterials-2108444-supplementary.pdf]

Supplementary Materials to:

Raman Study of the Diamond to Graphite Transition Induced by the Single Femtosecond Laser Pulse on the (111) Face

Andrey A. Khomich [1], Vitali Kononenko [1,2], Oleg Kudryavtsev [1,2], Evgeny Zavedeev [2] and Alexander V. Khomich [1,*]

[1] Kotelnikov Radio-Engineering and Electronics Institute of the Russian Academy of Sciences, Vvedensky Sq. 1, 141190 Fryazino, Russia
[2] Prokhorov General Physics Institute of the Russian Academy of Sciences, Vavilov St. 38, 119991 Moscow, Russia
* Correspondence: alex-khomich@mail.ru

1. XRD analysis of diamond plate.

Crystal orientation of diamond sample under study was characterized at x-ray double-crystal diffractometer TRS-K. A tube with a molybdenum anode used as an X-ray source (17.48-keV, λ = 0,7093 Å). The beam from the source and silicon 110 monocrystal monochromator was configured in reflection 220. The intrinsic half-width of the diffraction reflection of the monochromator is 2.1708 arcsec. Bragg angle of the monochromator θ_M = 10.644°.

The studied natural diamond sample had the shape of a spinning triangle with a side length of ~4 mm and a thickness of ~ 0.52 mm. The lattice parameter of diamond is 3.5668 A. The reflection of 111 diamond has a Bragg angle θ_S = 9.917°, the half-width of the intrinsic curve of diffraction reflection is 2.1108 arcsec. The theoretical extinction depth of this reflection is 2.24 μm. The aperture size of the slot in front of the sample was 0.1×4 mm^2. The beam projection on the sample was 0.6×4 mm^2. The projection determines the locality of the measurement on the surface of the sample. Thus, the full surface of the sample was illuminated. The mowing angles were determined in two mutually perpendicular directions.

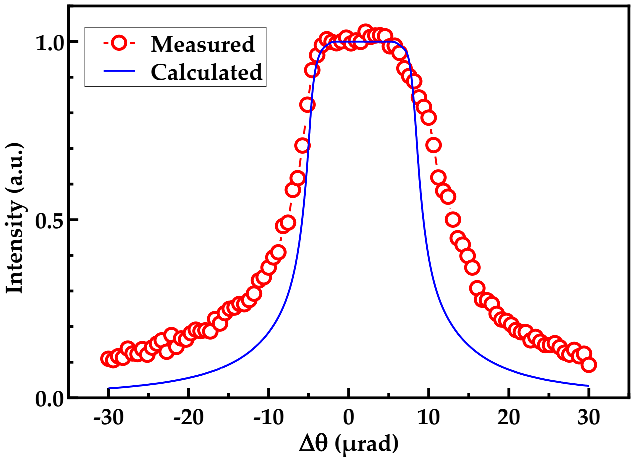

Figure S1. Rocking curve of the diamond crystal used for TRS-K double-crystal monochromator.

The peak width of the rocking curve depends on the quality of the surface of the samples - the presence of a damaged layer and the possible presence of defects in the volume. The asymmetry of the rocking curve is most likely caused by edge effects. The half-width of the peak was 19.9±0.5 arcs. The skews of the vector 111 with respect to the normal to the sample surface are -2.7±0.5° and 2.5±0.5°.

2. Photoluminescence spectra

Photoluminescence spectra (as a Raman spectra) were measured at the room temperature using a NanoFinder High End spectrometer (LOTIS TII Tokyo Instruments) combined with a three-dimensional scanning confocal microscope. Solid state laser with a wavelength of 473 nm was used for excitation. The laser radiation was focused on the surface of the samples with a 100x objective to the spot about 1 mm in diameter.

In addition to the diamond peak of the first and second order, the photoluminescence spectra (Figure S2) contain only a low-intensity band with a maximum near 492 nm, the nature of which has not been established. In this region of the spectrum in natural diamonds

of type 2a, an intrinsic effect can be observed, possibly containing vacancies [24] or a defect of interstitial-vacancy nature. Zero-phonon line at 492 nm was also observed in the spectra of CVD diamonds, where it was interpreted as a defect formed as a result of diamond

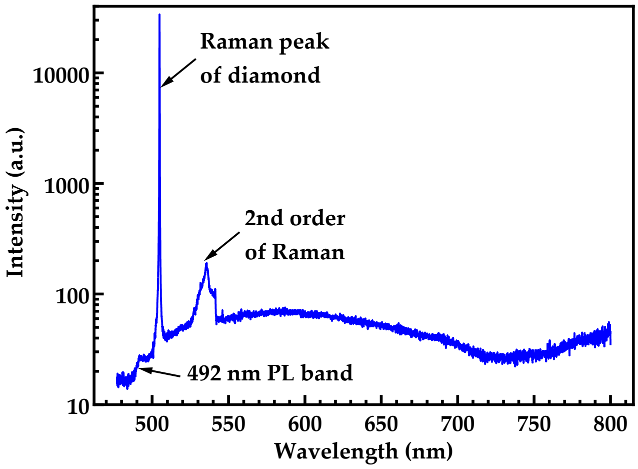

irradiation.

Figure S2. Photoluminescence spectrum of the natural diamond (111) plate under study.

3. Graphitization depth

The simplest way to estimate a distance, which the graphitization wave passes from the surface into the diamond bulk, based on the assumption that graphitization threshold F_{th} is a fixed value. Diamond-graphite transition occurs if the local laser fluence exceeds F_{th}, and otherwise diamond remains pristine. Laser radiation is absorbed when penetrating through the diamond sample along z axes thus the laser fluence steadily depletes. For two-photon absorption the energy losses are given as

$$\mathrm{dF} = \frac{\beta \cdot F}{\tau} F\mathrm{dz}, \tag{S1}$$

where τ is the pulse duration and $\beta \approx 10^{-9}\mathrm{cm/W}$ is the two-photon absorption coefficient of diamond. Therefore, the laser fluence depletion follows a relation:

$$\frac{1}{F(z)} = \frac{1}{F(0)} - \frac{\beta}{\tau} z. \tag{S2}$$

The $F(z)$ value decreases until achieves F_{th}. At this $z = D(F)$ point the graphitization stops and a depth of the graphitization wave penetration equals:

$$D(F) = \frac{\tau}{\beta}\left(\frac{1}{F^{\mathrm{th}}} - \frac{1}{F(0)}\right). \tag{S3}$$

As $F(0)$ is just the original laser fluence on the target surface we designate it as just F. And the previous relationship can be rewritten in the next form:

$$D(F) = \frac{\tau}{\beta}\left(\frac{1}{F^{\mathrm{th}}} - \frac{1}{F}\right). \tag{S4}$$

4. Determining the depth of craters and the thickness of the graphitized layer

The surface relief was investigated by white light optical profilometer (New View 5000, Zygo Corp.) after oxidizing the sample in an oven (600 °C) and removing the sp· hybridized phase from the bottom of the craters. The error of measuring the surface-profile height was less than 1 nm, which allowed us to reliably detect the variation in the diamond plate surface topology at the microcraters caused by laser graphitization. The depth of the crater formed by a laser pulse with an energy of 0.75 μJ in the central part of the crater is 70-72 nm (Figure S3a). Figure S3b shows the Raman spectra measured in different regions on the surface of the graphitized layer diamond surface and from. On the Figure S3a the circles show the projections of the measuring points of the Raman spectra onto the bottom of the crater.

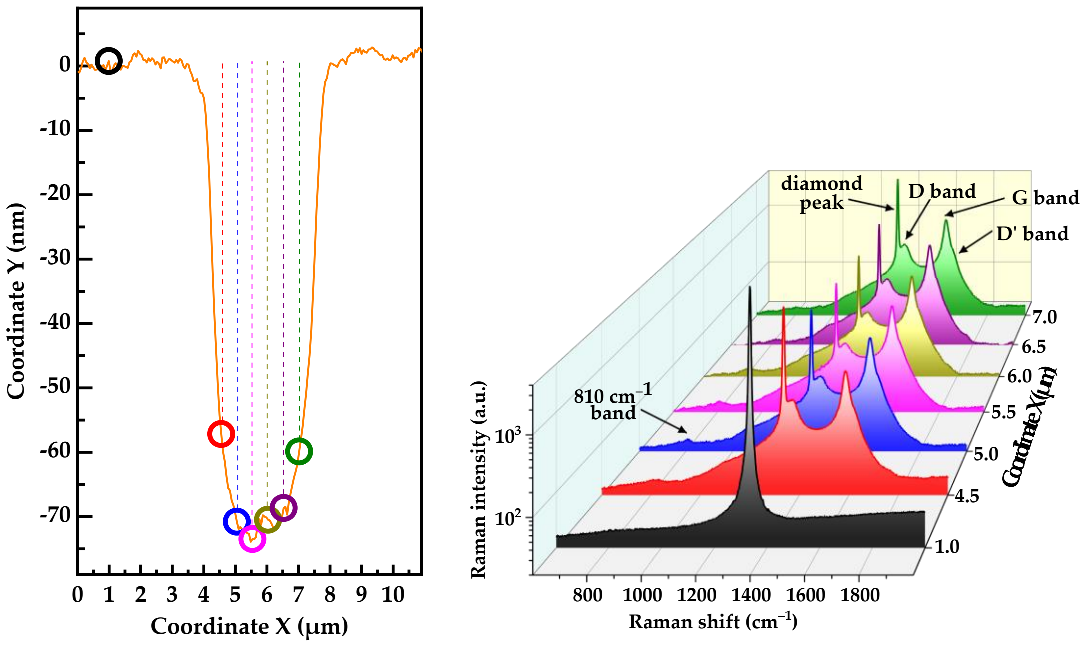

(**a**) (**b**)

Figure S3. (**a**) Cross profile of an ablation crater with a graphitized structure obtained at a laser pulse energy of 0.75 μJ after removal of the sp^2 phase. The dots indicate the projections of the spots where the Raman spectra were recorded. (**b**) Raman spectra at various points of graphitized layer obtained at a laser pulse energy of 0.75 μJ. The colors of the spectra correspond to the color of the dots in figure (b).

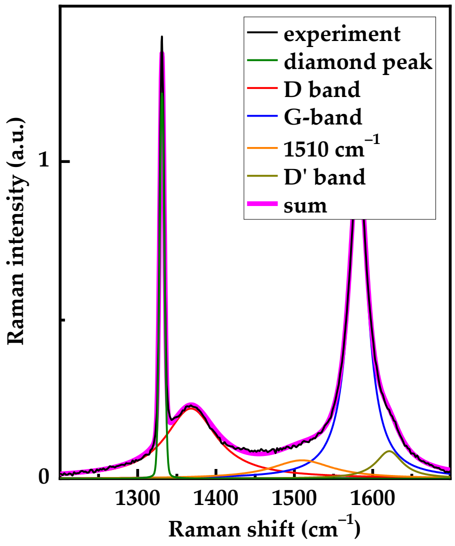

Figure S4. An example of the decomposition of the Raman spectrum with Voigt profile of diamond peak.

The thickness of graphitized layer can be calculated from the decrease of intensity of a diamond line because of the signal absorption in it according to the Beer-Lambert law:

$$I(l) = I_0 e^{-\mu l} \qquad (S5)$$

where I is the integral intensity of the Raman diamond peak (Figure S4), l is the thickness of graphitized layer and μ is the optical absorption coefficient. The integral intensity of the Raman diamond peak was determined by approximating the spectrum with Pseudo-Voigt profile. According to the results from [29], nanocrystalline graphite layer at the surface of laser-graphitized diamond has $\mu = 1.16\times10^5$ cm^{-1}.

The decrease of the intensity of diamond line results first from the loss of intensity of probe laser and second from the loss of scattering signal. That is why we need to divide the value of l by 2 in the estimation of the thickness of absorbing layer:

$$l = \frac{ln\left(\frac{I'_d}{I_d}\right)}{2\mu}, \qquad (S6)$$

where I_d and I'_d are the intensities of diamond line from origin plate and from irradiated surface correspondingly.

It should be noted that the determination of the thickness of the graphitized layer on diamond by the attenuation of the amplitude of the diamond Raman peak is a qualitative rather than a quantitative method and makes it possible to compare the thicknesses of layers formed under similar technological conditions. One of the main problems here is the possible depth inhomogeneity of the graphitized layer and the dependence of the optical properties of the graphitized layer on the structure and specific density of the graphitized material. Also, the results of measurements near the edges and on the slopes of craters should be treated with caution, since the size of the Raman laser beam and the dimensions of the crater differ only by a few times, so the Raman spectrum is formed in the region of the crater, where the thickness of the graphitized layer can change.

In our work, with a laser fluence of the order of 4-6 J/cm^2 (spots 5.0÷6.5 μm at Figure S3), HOG is formed on the diamond surface, the optical properties of which are closer to those of pyrolytic graphite. The optical absorption coefficient of pyrolytic graphite at 500 nm is μ = 1.67×10^5 cm^{-1} [31] or about 40% higher than that used in the calculations in Figure 3 in the manuscript. Accordingly, the calculated value of the graphite thickness will be up to 40% less, that is, about 90-100 nm. This value agrees well with the crater depth of 70–75 nm (Figure S3a), taking into account the transformation of diamond into graphite, which has a specific density approximately 1.5 times lower than diamond.

References

24. Zaitsev, A.M.; Kazuchits, N.M.; Moe, K.S.; Butler, J.E.; Korolik, O.V.; Rusetsky, M.S.; Kazuchits, V.N. Luminescence of brown CVD diamond: 468 nm luminescence center. *Diam. Relat. Mater*. **2021**, *113*, 108255.
29. Komlenok, M.S.; Dezhkina, M.A.; Khomich, A.A.; Orekhov, A.S.; Konov, V.I. Measuring the local thickness of laser-induced graphitized layer on diamond surface by Raman spectroscopy. *Phys. Stat. Sol. B* **2019**, *256*, 1800686.
31. Querry, M.R. Optical Constants, Contractor Report, CRDC-CR-85034; Missouri University: Columbia, MO, USA, 1985.
